# The Basal Forebrain Modulates Neuronal Response in an Active Olfactory Discrimination Task

**DOI:** 10.3389/fncel.2020.00141

**Published:** 2020-06-05

**Authors:** Alexia Nunez-Parra, Christian A. Cea-Del Rio, Molly M. Huntsman, Diego Restrepo

**Affiliations:** ^1^Department of Cell and Developmental Biology, Rocky Mountain Taste and Smell Center and Neuroscience Program, University of Colorado Anschutz Medical Campus, Aurora, CO, United States; ^2^Department of Biology, Faculty of Science, Universidad de Chile, Santiago, Chile; ^3^Department of Pharmaceutical Sciences, Skaggs School of Pharmacy and Pharmaceutical Sciences, University of Colorado Anschutz Medical Campus, Aurora, CO, United States; ^4^Centro de Investigacion Biomedica y Aplicada (CIBAP), Escuela de Medicina, Facultad de Ciencias Medicas, Universidad de Santiago de Chile, Santiago, Chile; ^5^Department of Pediatrics, School of Medicine, University of Colorado Anschutz Medical Campus, Aurora, CO, United States

**Keywords:** attention, go/no–go, *in vivo*, anticipation, discrimination, acetylcholine

## Abstract

Successful completion of sensory decision-making requires focusing on relevant stimuli, adequate signal/noise ratio for stimulus discrimination, and stimulus valence evaluation. Different brain regions are postulated to play a role in these computations; however, evidence suggests that sensory and decision-making circuits are required to interact through a common neuronal pathway to elicit a context-adequate behavioral response. Recently, the basal forebrain (BF) region has emerged as a good candidate, since its heterogeneous projecting neurons innervate most of the cortical mantle and sensory processing circuits modulating different aspects of the sensory decision-making process. Moreover, evidence indicates that the BF plays an important role in attention and in fast modulation of neuronal activity that enhance visual and olfactory sensory perception. Here, we study in awake mice the involvement of BF in initiation and completion of trials in a reward-driven olfactory detection task. Using tetrode recordings, we find that BF neurons (including cholinergics) are recruited during sensory discrimination, reward, and interestingly slightly before trial initiation in successful discrimination trials. The precue neuronal activity was correlated with animal performance, indicating that this circuit could play an important role in adaptive context-dependent behavioral responses.

## Introduction

Efficient sensory decision-making in a constantly changing environment requires neuronal circuits to be plastic and rapidly modify their activity. Cortical and subcortical processing are influenced substantially by feedback and neuromodulatory afferents eliciting experience-induced modulation of neuronal excitability lasting from milliseconds to hours (Hasselmo and Giocomo, 2006; Citri and Malenka, 2008; Picciotto et al., 2012; Sara and Bouret, 2012; Avery and Krichmar, 2017). It has been suggested that simultaneous neuromodulation of neural circuits that process sensory, cognitive, and motor information is required to maintain neuronal dynamics for proper decision-making (Grossberg et al., 2015). Therefore, the brain region(s) acting as a modulator should innervate most if not all sensory processing regions. In addition, the brain region should exhibit the ability to modulate neuronal excitability dynamically allowing rapid context-dependent changes in information processing to elicit adequate behavioral outputs. The basal forebrain (BF) emerges as a good candidate to participate as an integrator and neuromodulator source for behavior since it is one of the most important and widely projecting neuromodulatory circuits in the mammalian brain (Gritti et al., 2006) reaching the entire cortical mantle, hippocampus, and the olfactory system among others (Luskin and Price, 1982; Zaborszky et al., 1986; Zaborszky, 2012). Functionally, it has been linked with attention (Klinkenberg et al., 2011), arousal (Buzsaki et al., 1988), and learning and memory (Everitt and Robbins, 1997; Klinkenberg et al., 2011). Specifically, its subnuclei have been proposed to play important roles in components of goal-directed behaviors such as motivational saliency (Lin and Nicolelis, 2008), sensory discrimination (Lin and Nicolelis, 2008; Devore and Linster, 2012; Nunez-Parra et al., 2013; Pinto et al., 2013; Devore et al., 2016), and cortical control (Picciotto et al., 2012).

The wide array of neurophysiological and cognitive functions that the BF is involved in correlates with the neuronal complexity found in the region. Among the variety of neuronal types found in the BF (Zaborszky and Duque, 2000), the cholinergic corticopetal projecting neurons have been extensively studied due to the important and dense top–down coordination role acetylcholine plays in cognitive functions such as attention. This idea arose from studies where pharmacological blockade or selective lesions of cholinergic neurons (CNs) in BF produced impairments in attention, memory, and operant conditioning performance (Turchi and Sarter, 1997; Curzon et al., 1999; McGaughy et al., 2000; Klinkenberg et al., 2011; Devore and Linster, 2012; Picciotto et al., 2012; Luchicchi et al., 2014; Dannenberg et al., 2015; Rangel et al., 2016). Moreover, in attention-demanding tasks, cholinergic release enhances cue detection and sensory discrimination (Sarter et al., 2005).

Here, we ask whether BF neurons are involved in the decision-making process in a non-cued olfactory-based self-initiated task. We addressed this question by recording the neural activity of the BF while the animals were freely engaged in a go/no–go task with voluntary trial start. Moreover, using optogenetic tagging, we identified CNs among the recorded units offline.

## Results

### The Firing Rate of Basal Forebrain Neurons Changes Before Initiation of the Trial

**FIGURE 1:**
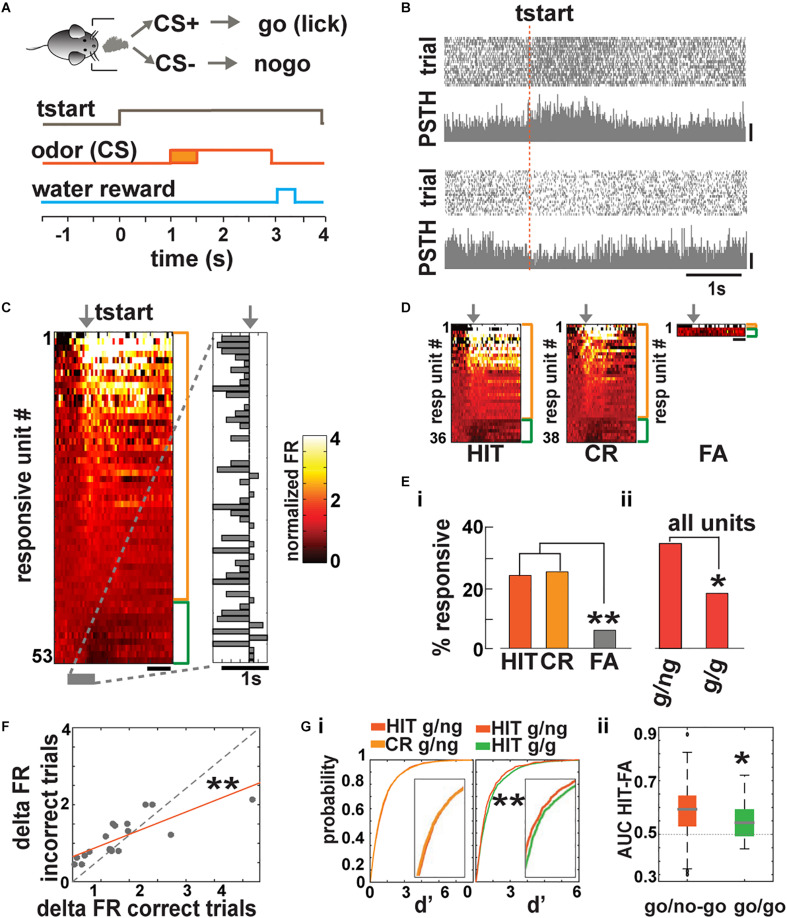
Basal forebrain (BF) neuronal activity is recruited during trial initiation in a go/no–go task. **(A)** The odor is delivered 1.5 ± 0.5 s after the mouse starts the trial. In response to CS^+^, the animal must lick at least once in four 0.5-s segments to receive a water reward. **(B)** Spike scatterplot for two BF single units in the go/no–go task for a mouse performing >80% correct responses. FR increased for one unit (top; scale bar, 50 Hz) and decreased for the other (bottom; scale bar, 20 Hz) during trial initialization (tstart). **(C)** Heat map depicting the normalized mean firing rate of all responsive units aligned by tstart (arrow; side bars, orange: FR increase, –43/153– total; green: FR decrease, –10/153–; scale bar, 1 s). A unit is classified as responsive if there is a statistical difference between the FR after the animal entered the port compared to the FR of that unit before the animal entered the port (*p* < *p*FDR; *p*FDR, the FDR critical significance level per animal, ranged from 0.006 to 0.03; *n* = 153 units, 141 single units, and 12 multiunits from 8 mice and 10 sessions). Right, bar graph showing the time point where the FR changed ±2 standard deviations (SD) from the mean. **(D)** Heat maps depicting the normalized FR of responsive units sorted by correct responses (hit and correct rejection, CR) and incorrect trials (false alarms, FA; scale bar, 1 s). We did not find miss responses. **(E)** Percent of responsive cells for FR aligned to tstart sorted by behavioral outcome and task. A larger number of neurons **(i)** respond to correct responses (HIT and CR) when compared to incorrect responses (FA, go/no–go HIT and CR different from FA, chi-squared test *p*FDR = 0.05, **pHIT vs FA = 0.001, **pCR vs FA = 0.0008) and more units **(ii)** were recruited during the go/no–go task compared to the go/go (chi-squared test *p*FDR = 0.05, *ptotal = 0.03; g/ng, go/no–go; g/g, go/go). **(F)** The change in FR during tstart is significantly different between correct and incorrect trials (ANCOVA, *F* = 16.6, Tukey’s *post hoc*, **p = 0.0004). **(G)** Left, cumulative probability function of *d*’ for all the recorded units. The curves were not different between correct responses (HIT and CR) in the go/no–go task but were different between HIT in the go/no–go and go/go (**KS test *p* = 3.6 × 10^−8^). Right, whisker plot for the area under de curve (AUC) of each unit in ROC space. Units acted as better classifiers in the go/no–go test compared to the go/go *t*-test, *p = 0.03.

To study the dynamics of recruitment of BF neurons in animals engaged in a self-initiated decision-making task, we implanted a multielectrode device in the horizontal diagonal band of Broca/magnocellular preoptic (HDB/MCPO) nuclei and proximity in the BF of trained adult mice (Supplementary Figures S1A,B). We recorded from HDB/MCPO because these are the only BF nuclei that send projections to the olfactory bulb and olfactory cortex (Zaborszky et al., 1986). Animals were trained in a go/no–go olfactory discrimination associative learning task (Slotnick and Restrepo, 2005). This task studies the ability of a thirsty rodent to lick to obtain water in response to a rewarded conditioned stimulus (CS^+^) and refrain from licking in response to an unrewarded stimulus (CS^–^) (Figure 1A). The CS^+^ and CS^–^ were odors randomly chosen from an odor set known to elicit neuronal response in the olfactory system (Doucette et al., 2011) (see section “Materials and Methods”). Each trial is self-initiated 1–1.5 s after the computer detects the mouse entering the odor port.

We recorded neuronal activity during 200 trial sessions in animals proficient in differentiating between the two odorants (percent of correct responses, ≥80%). We found that single units responded with increases or decreases in firing rate (FR) during trial initiation (tstart). Figure 1B shows examples of scatterplots of spike firing and peristimulus histograms (PSTHs) for two single BF units aligned to trial initiation (tstart) (top, increase in FR; bottom, decrease in FR).

We analyzed the time course of FR changes aligned to tstart in 153 units total. A unit was categorized as responsive if the changes in FR assessed for 1 s after tstart were statistically significantly different from the basal FR assessed 1.3–0.3 s before tstart tested with a paired *t* test corrected for multiple comparisons using the false discovery rate (FDR) (Curran-Everett, 2000). We choose this time range to calculate the basal FR, since there appeared to be a change in FR just before the animal entered the port (see below). We found that a substantial fraction of BF neurons (53 out of 153 or 34.6%) exhibited significant increases (43 out of 153 or 28.1%) or decreases (10 out of 153 or 6.5%) in FR when the animal initialized a trial (*p* < *p*FDR, *p*FDR, the FDR critical significance level per animal, ranged from 0.006 to 0.03; *n* = 153 units, 141 single units and 12 multiunits from 8 mice and 10 sessions). Figure 1C shows on the left side a heatmap illustrating the FR time course for the 53 units that were significantly responsive and on the right side the time when the FR changed by 2 × SD above or below basal FR. Interestingly, for most of the units, the change in FR took place before the animal entered the odor port (mean onset time −260 ms with a 95% bootstrapped confidence interval ranging from −168 to −350 ms) suggesting that BF neurons are involved in behavioral functions associated with trial preparation and anticipation.

We found that the number of BF neurons that exhibited a significant change in FR at the start of correct response trials (hits, 36 out of 149 units, −29 or 19.5% increase and 7 or 4.7% decrease their FR; and correct rejections, CR, 38 out of 150 total units recorded on those trials, −30 or 20% increase and 8 or 5.4% decrease their FR) is larger than the number of responsive units in false alarm trials (FA, licking in response to the CS^–^; 4 of 67 total units recorded during FA trials were responsive, 1 unit or 1.5% increase and 3 or 4.4% decrease their FR, Figures 1D,E). A chi-squared test indicated that the difference in the number of responsive units between FA trials and correct trials is significant (*p* < *p*FDR = 0.05). This shows that engagement of BF neurons during the precue epoch reflects the behavioral outcome of the trial, suggesting that activity of these neurons may play a role in successful discrimination.

To further test the relationship between BF neural activity at trial initiation and behavioral outcome, we asked whether the change in BF neuronal activity is affected by engagement in sensory discrimination of rewarded vs. unrewarded odorants. We recorded neuronal activity of animals trained in a go/go task where the mouse is rewarded randomly for 70% of the trials regardless of the identity of the odorant. The key difference with go/no–go is that, in the go/go task, both odorants are rewarded and no sensory discrimination is required to receive the reward. We found that the number of units responsive at port entry was significantly lower in the go/go task compared to the go/no–go task (Figure 1Eii and Supplementary Figure S2, chi-squared test *p* < *p*FDR = 0.05, go/no–go = 53 responsive out of 153 or 34.6%), go/go = 8 responsive out of 44 or 18.2%, all of which increased their FR, suggesting that BF neuronal recruitment before trial initiation may play a role in adequate stimulus discrimination. In the go/go task, the units that responded to trial initialization also exhibited a change in FR before the animal entered the port (mean onset time −175 ms with a 95% bootstrapped confidence interval ranging from 167 ms to −517 ms). We also compared the change in FR after the start of the trial between units that responded to correct and incorrect trials. Figure 1F shows the relationship between change in FR for incorrect trials and the change in FR for correct trials. The data are fit with a line with a slope significantly smaller than one, suggesting that engagement of BF neurons reflects correct behavioral performance (Figure 1F; ANCOVA, *F* = 16.6, Tukey’s *post hoc*, *p* = 0.0004, *n* = 19). To further determine whether recruiting BF neurons during trial initiation relates to animal behavior, we calculated *d*′, defined as the difference in the change in FR upon trial initiation normalized by the standard deviation of basal FR (*d*′) per trial. Figure 1Gi shows no difference for *d*′ for hit vs. CR for the go/no–go task (Figure 1G, KS test *p* = 0.11, *n* = 6,289 trials for hit and 6,649 responsive units for CR). In contrast, Figure 1Gii shows a significant difference in *d*′ curves of hit trials in the go/no–go task vs. hit trials in the go/go task (KS test *p* = 3.6 × 10^–8^, *n* = 1,266 trials units for go/go). These data indicate that neurons responded similarly during correct responses that required sensory discrimination but responded differently when no discrimination was required. To study how effective the change in FR during trial initiation is at classifying correct vs. incorrect behavioral outcome, we used the receiver operant characteristic (ROC) analysis (Fawcett, 2006) and measured the area under the curve (AUC) for each unit. The higher the AUC (maximum 1), the better the unit differentiates between correct and incorrect responses (AUC of 0.5 indicates no differentiation). We found that units in the go/no–go task were a more effective classifier than in the go/go scenario (Figure 1Gii, *t* test *p* = 0.03, *n* = 153 for go/no–go and 44 for go/go), suggesting that BF neuron activity is related to adequate decision-making in sensory discrimination.

### Basal Forebrain Neurons Exhibit Changes in Firing Rate When Conditioned Stimulus or Reinforcement Are Delivered

**FIGURE 2:**
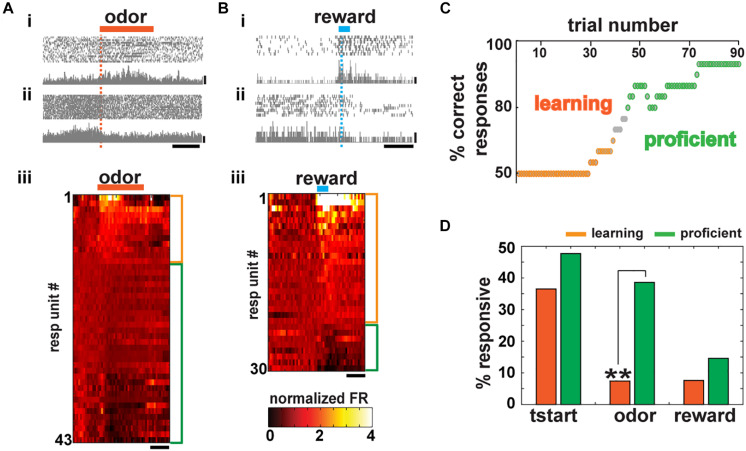
Basal forebrain (BF) neurons are recruited during conditioned stimulus and reward presentation and its number increases after learning. **(A)** Spike scatterplot for two BF units in the go/no–go task for a mouse performing >80% correct responses. Firing rate (FR) increased for the unit at the top (i) decreased for the unit in the bottom (ii) during odor delivery (orange bar; scale bar, 50 Hz). (iii) Heat map depicting the normalized mean firing rate of all responsive units aligned by odor presentation (side bars, orange: FR increase, –12/153–; green: FR decrease, –31/153–; paired *t* test corrected for multiple comparisons, *p* < *p*FDR, *p*FDR: 0.02–0.01 per mouse). Units are sorted by the change in FR. Scale bar, 1 s. **(B)** Basal forebrain unit activity aligned to water delivery (reinforcement). As in **(A)**, some units increase (i) and others decrease (ii) their FR (scale bar, 20 Hz). Responsive units exhibit a statistically different FR post–pre water delivery (side bars, orange: FR increase, -22/148-; green: FR decrease, -8/148-; paired *t* test corrected for multiple comparisons, *p* < *p*FDR, *p*FDR: 0.003–0.03 per mouse) (scale bar 1 s). **(C)** Representative learning curve of an animal during the first training session in the go/no–go task. **(D)** Percent of responsive cells during task learning (percent of correct responses = 50%) and when the task has already been learned (proficient, percent of correct responses ≥80%). There is no statistical difference in the number of neurons that change their activity after trial initialization and reward presentation, but a significant increase in the number of BF neurons recruited during odor presentation after learning (chi-squared test *p*FDR = 0.02, *p* tstart = 0.37, *p odor = 0.004, *p* reward = 0.039).

To determine whether BF neuronal activity is recruited during other epochs of the behavioral trial in our experimental design, we aligned the normalized FR of the recorded units either to delivery of the conditioned stimulus (CS or odor) or the reward. Figure 2 shows changes in FR aligned to odor delivery (Figure 2A) or reward delivery (Figure 2B) for mice performing >80% in the go/no–go task. As described in previous studies (Lin and Nicolelis, 2008; Thomson et al., 2014; Devore et al., 2016), we found that neurons either increase (Figures 2Ai, iii; 12/153 or 7.8%) or decrease (Figures 2Aii, iii, 31/153 or 20.3%), their FR in response to the stimulus. Specifically, 21 out of 150 units (14%) recorded were recruited during CS^+^ delivery and 45 out of 150 (30%) during CS^–^ (*t* test *p* < *p*FDR, *p*FDR per session ranged from 0.02 to 0.01, 0.003 to 0.04, 0.02 to 0.01 for odor, CS^–^ and CS^+^ and reward, respectively). As shown in the heat map of Figure 2Aiii, some units responded transiently, while others responded with slow sustained changes in FR.

In addition, units were also recruited during water (reward) delivery (22/148 or 14.9% increase and 8/148 or 5.4% decrease their FR, Figure 2B, *t* test *p* < *p*FDR, *p*FDR per session ranged from 0.003 to 0.03). Units that responded to reward in this self-initiated task also tended to respond to the conditioned stimulus as described by Lin and Nicolelis (2008) in rodents trained in a cue-oriented task (Supplementary Figure S2D).

Supporting the idea that the BF could play a role in stimulus discrimination and reward association, the percent of units that responded during odor or reward delivery in the go/go task (when regardless of the odorant 70% of the trials are rewarded) are significantly smaller than the responses in the go/no–go task (28.1% go/no–go vs. 9.1% go/go and 20.3% go/no–go vs. 4.8% go/go during stimulus presentation and water delivery, respectively, Supplementary Figures S2B,C; chi-squared test, *p* < *p*FDR = 0.05, two pairwise comparisons).

### Basal Forebrain Neurons Become More Responsive to the Stimulus as the Animal Learns to Differentiate Odorants in the Go/No–Go Task

Our data suggest that neurons from the BF are required for adequate decision-making and stimulus discrimination in proficient animals, raising the question whether the number of neurons coding for information during the different epochs of the behavioral trial increased as the animal learned to discriminate between rewarded and non-rewarded odors. We compared the change in FR during the different epochs of the trial when the animal was learning to discriminate (= 50% correct trials) and when the animal was proficient in their response to the rewarded odorant (>80% correct trials). A representative learning curve is shown in Figure 2C. It starts with 50% correct responses, while the mouse gradually becomes proficient until reaching criteria (>80% correct responses, hits and CRs) within a session. We observe that the number of BF-responsive neurons during trial initialization does not increase as the animals learn to associate the stimulus with the reward (Figure 2D, chi-squared test *p* = 0.37 > *p*FDR = 0.016, 10 out of 27 units or 37% were responsive during learning and 21 out of 44 or 47.7% were responsive when the animal was proficient). These units are likely engaged during the instrumental shaping of the task that occurs before animals are trained in the go/no–go task and could reflect the motivation and initial attention required to start the trial.

After that initial training, animals are trained to lick in response only to the rewarded stimulus that has no hedonic value at the beginning of the session. The number of responsive units when FR is aligned to odorant onset increased dramatically with learning (Figure 2D, 7.4 vs. 38.6%; chi-squared test, *p* = 0.004 < *p*FDR = 0.016). In contrast, for the reward epoch, we found no statistical difference between the number of responsive neurons before and after the animal became proficient (7.6 vs. 14.6%; chi-squared test, *p* = 0.39 > *p*FDR = 0.016). Taken together, our results suggest that BF neurons play a role in actively engaging the animal in the task (trial start epoch), in correct odorant discrimination (odor epoch), and responding to the reward (reward epoch) and that learning increases the number of neurons engaged in the odor epoch.

### A Subset of the Basal Forebrain Cholinergic Neurons That Are Responsive During Trial Initiation or Conditioned Stimulus Epochs Are Cholinergic

**FIGURE 3:**
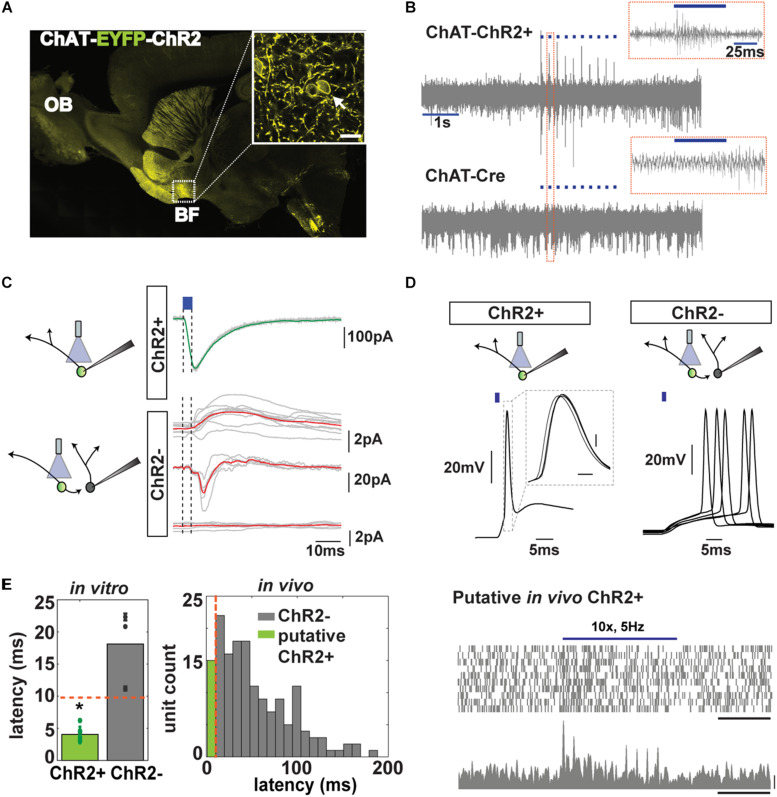
Cholinergic neuron (CN) optotagging in the basal forebrain (BF). **(A)** Confocal EYFP fluorescence for a sagittal brain section of a ChAT-EYFP-ChR2 mouse (inset: BF at 63× magnification, bar 10 μm); arrow: fluorescence along the membrane; OB, olfactory bulb). **(B)** Light pulses (50 ms, blue trace) increase the extracellular spiking activity of neurons in ChAT-ChR2 animals but not in ChAT-Cre controls (inset: response to one light pulse). **(C)** Representative traces of *in vitro* voltage clamp recordings of a ChAT-ChR2^+^ neuron (top) and ChR2^–^ neuron (bottom) after light stimulation (blue). Red trace shows the average response. **(D)** Representative traces of *in vitro* whole cell current clamp recordings of ChAT-ChR2^+^ (*n* = 7, 7/7 responded to 1 ms Lstim) and ChR2^–^ neurons (*n* = 9, 3/9 responded to Lstim). Notice the jitter of the response of the synaptically connected ChR2^–^ neuron. **(E)** Left, latency of light activation of ChR2^+^ (4.1 ± 0.4 ms) and ChR2^–^ neurons (18.1 ± 3.5 ms, *t* test, *p < 0.001). Red line: criterion for a neuron to be considered cholinergic. Right, latency histogram of all neurons recorded *in vivo* (green: latency <10 ms). **(F)** Scatter plot (top, 20 trials, bar, 1 s) and peristimulus time histogram (PSTH) (bottom, bars, 1 s and 20 Hz) of an identified cholinergic neuron to 10 pulses of a light stimulation at 5 Hz (see criteria in the text).

The neuronal makeup of the BF is heterogeneous with glutamatergic, GABAergic, and cholinergic projection neurons, among others (Duque et al., 2007; Zaborszky, 2012). The cholinergics have granted particular attention since they project to the whole cortical mantle and actively participate in cortical plasticity (Conner et al., 2010) and sensory processing (Linster et al., 2001; Pinto et al., 2013). This motivated us to determine whether BF CNs were responsive in any epochs of our self-initiated task in proficient animals. To identify CNs, we used optogenetic tagging (Hangya et al., 2015). We used mice expressing ChR2-EYFP under control of the choline acetyl transferase (ChAT) promoter. Once the behavioral session concluded, we delivered light stimulation (10 trials of 10 50-ms pulses at 5 Hz) through the optic fiber of the optetrode implanted in the BF of mice expressing ChR2-EYFP selectively in CNs (ChR2^+^ neurons, Figure 3A, see section “Materials and Methods”). The pronounced increase in spiking frequency in a subset of units was not observed in control ChAT-Cre animals (Figure 3B) regardless of the frequency of stimulation (1 or 5 Hz), discarding the possibility that light delivery could generate false spikes due to thermal stimulation (Grosenick et al., 2015).

The BF, however, exhibits intricate local circuitry with abundant cholinergic collaterals terminating in non-cholinergics (Duque et al., 2007), raising the possibility that light-responsive neurons *in vivo* might not express ChR2. We confirmed the local connectivity by performing *in vitro* whole-cell patch clamp recordings in acute brain slices from the BF. In the voltage clamp mode, we found that brief light stimulation always elicited inward currents in ChR2^+^ neurons (Figure 3C). In contrast, non-CNs (identified by their lack of ChR2-EYFP fluorescence, ChR2^–^ neurons) located in close proximity to a neuron expressing ChR2-EYFP (ChR2^+^) exhibited an array of responses after being transsynaptically activated by optogenetic activation of CNs. A small number of non-CNs (*n* = 1/10) exhibited an outward current after the cholinergic ChR2^+^ neurons were activated; some (*n* = 3/10) exhibited an inward current or a biphasic response (2/10), and most of them (*n* = 4/10) showed no change (Figure 3C).

To obtain information relevant to the correct identification of CNs *in vivo*, we studied the latency for light activation of cholinergic ChR2^+^ and non-cholinergic ChR2^–^ neurons through *in vitro* current clamp (Figure 3D). We found that there was a clear and significant difference in latency of responses between these neurons (18.1 ± 3.5 ms, *n* = 3/9 responded for ChR2^–^ vs. 4.1 ± 0.4 ms, *n* = 7/7 for ChR2^+^, respectively, *t* test, *p* < 0.001) allowing us to establish 10 ms as a cutoff for maximum latency for neurons that were directly activated by light (Figure 3E), in accordance with Hangya et al. (2015). Only three out of nine non-cholinergics exhibited action potential generation after activating neighboring ChR2^+^ neurons, while all nine ChR2^+^ neurons responded.

**FIGURE 4:**
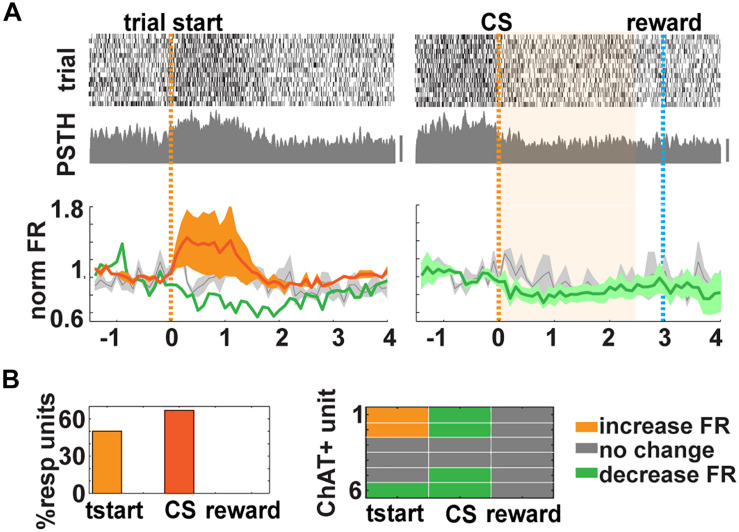
Cholinergic neurons (CNs) respond to trial start in the go/go–no (GNG) task. **(A)** Top and middle. Examples of responses for units classified as cholinergic. Top, raster plot of 15 trials aligned by trial start (orange dashed line, left) or conditioned stimulus (CS) presentation (right, light blue dashed line: water delivery or reward, bar 50 Hz). Middle, peristimulus time histogram (PSTH). Bottom, mean normalized firing rate (FR) for six identified CNs (increase: orange; decrease: green; no change: gray). Shaded area represents the SD of the mean. **(B)** Summary of CNs responses. Left, percent responding to tstart (66.7%), CS (83.3%), and reward (0%). Right, comparison of the CNs responses in all events.

*In vivo*, we found that 15 out of 186 units (from go/no–go and go/go tasks) exhibited latency <10 ms (Figure 3E). In addition, we used two other properties to classify a neuron as cholinergic: (1) it had to exhibit a statistically significant increase in FR after light stimulation in a paired *t* test with correction for multiple comparisons, and (2) it had to display a reliability of response of 100% (they had to spike within 200 ms after light stimulation in all 10 trials; Figure 3F and Supplementary Figures S3A–C). CNs, despite their wide and critical role in brain function, are sparsely distributed and account for only 5% of the BF neurons (Gritti et al., 2006). With our conservative criteria, we classified 6 out of 186 (3.2%) units as cholinergic in accordance with the numbers found in other studies (Lin and Nicolelis, 2008; Hangya et al., 2015). We found that three of these six optogenetically tagged CNs responded with a significant change in their FR when the mouse decided to enter the port (3/6 units or 50% of responsiveness, paired *t* test *p* < *p*FDR = 0.025; mean onset latency of FR change, 100 ms ± 100) and when presented with the CS (67% of responsiveness, paired *t* test *p* < *p*FDR = 0.033, Figure 4 and Supplementary Figure S3D). We did not find responses to reward in these six neurons (Figure 4 and Supplementary Figure S3E). Therefore, although CNs are sparse, yielding recording from a small number of units, the changes in FR are clear and consistent from trial to trial, indicating that these neurons are engaged in trial initiation and CS discrimination.

## Discussion

On the basis of *in vivo* electrophysiological recordings in freely moving and behaving animals, we demonstrated that neurons from the BF are engaged throughout the decision-making process in a goal-directed task. Transient changes in the activity of BF, specifically the HDB/MCPO and proximity, were found during trial initialization anticipating the stimulus, stimulus discrimination, and in reward association in the go/no–go odor discrimination task. Importantly, the number of units displaying changes in FR increased for the stimulus discrimination epochs as the animal learned to discriminate the odorants. Furthermore, the changes in FR were found to be related to correct outcome in the trial, and the number of units that displayed a change in FR decreased in a go/go task where animals receive reward regardless of the odorant, indicating that BF activity plays a role in correct outcome of the trial. Finally, through optogenetic tagging, we found that BF CNs are involved in this processes.

### Basal Forebrain and Anticipatory Activity

The capacity of the brain to correctly respond to environmental cues has been linked in recent years to its ability to predict future outcomes. The anticipatory behavior has been described to improve performance not only by enhancing motor preparedness and reaction time but also by improving perception (Nobre et al., 2007) and more efficiently processing the upcoming sensory input (Bastiaansen and Brunia, 2001; Jaramillo and Zador, 2011). Specifically, baseline rates of neurons in HDB/MCPO BF has been shown to be higher during the acquisition phase of an odor–reward association than during spontaneous investigation or the recall phase of an odor reward association (Devore et al., 2016). Furthermore, neurons in other nucleus of the BF, the nucleus basalis, responded before stimulus onset and continued for seconds after reward delivery in a whisker-dependent tactile discrimination two-alternative forced choice task (Thomson et al., 2014). Interestingly, Thomson et al. (2014) observed that the anticipatory modulation in neuronal FR began ∼1 s before the onset of the mechanical deflection of the whiskers, similar to our results, where we observed anticipatory changes in FR of the BF before the animal enters the odor port. They hypothesized that neurons of the nucleus basalis participated in the circuit defining animal’s expectations in the task (Thomson et al., 2014). In addition, neuronal responses with onsets before the first lick were reported in the olfactory tubercle in a self-initiated water-motivated dry lick instrumental task (Gadziola and Wesson, 2016) and an intermodal selective attention task (Carlson et al., 2018). They found that neurons of the olfactory tubercle fired in anticipation of the expected reward probably to invigorate instrumental training in states of reduced motivation. Finally, in primates, the anticipatory activity of neurons in the caudate nucleus correlates with reward association, expectation, and response latency, probably reflecting the animal’s motivational state (Lauwereyns et al., 2002; Watanabe and Hikosaka, 2005).

In conclusion, there is evidence of anticipatory neuronal activity in different brain regions, suggesting that neuronal activity linked to expectation might play an important role in behavior.

Interestingly, we also found that this anticipatory activity was correlated with behavioral performance, supporting an additional role of early neuronal activity in attaining correct stimulus discrimination. This idea follows the line of evidence suggesting that top–down modulation might be as important as the external stimulus information in sensory processing and perception in the visual system (Gilbert and Sigman, 2007) or other sensory-motor modalities, like the tongue–jaw motor cortex, which anticipatory prestimulus activity can be predictive of licking direction in a somatosensory detection task (Mayrhofer et al., 2019). A recent article found that inhibition of the neuronal activity of the BF using Designer Receptors Exclusively Activated by Designer Drugs (DREADDs) interrupted the ability of rats in increasing their discrimination accuracy in a sustained attention task in response to a high reward probability trials (Tashakori-Sabzevar and Ward, 2018), further suggesting that the BF could play a role in sensory discrimination. In humans, electroencephalogram (EEG)/magnetoencephalogram (MEG) studies have suggested that anticipatory attention could promote desynchronization of oscillatory brain activity (Bastiaansen and Brunia, 2001; Rohenkohl and Nobre, 2011), which would enhance perception. Future studies with electrophysiology or imaging of neural activity imaging are necessary to determine the role of neuronal dynamics of the BF in sensory discrimination during reward expectation.

### Cholinergic Neurons and Anticipatory Activity

As mentioned before, the neuronal population of the BF is an intricate heterogeneous network with glutamatergic, GABAergic, and cholinergic projection neurons, among others (Duque et al., 2007; Zaborszky, 2012). Using optogenetic tagging, we identified CNs from our recorded units and found that the activity of CNs were also engaged and modulated during trial initialization, which could participate in the preparation of the decision-making process.

The role of slow changes in ACh concentration, in time scales from minutes to hours, is well established based on the finding that CNs are recruited during arousal (Buzsaki et al., 1988) and that ACh is slowly released and diffuses through the cortical mantle (Munoz and Rudy, 2014). However, recent evidence suggests that fast transient changes (milliseconds to seconds) in ACh may regulate neuronal processes affecting decision-making and behavioral performance in instrumentally cued tasks (Parikh et al., 2007; Lin and Nicolelis, 2008; Pinto et al., 2013; Munoz and Rudy, 2014; Hangya et al., 2015; Gritton et al., 2016). Here, we found that, in a more naturalistic scenario, where the cholinergic system is not permanently engaged, such as a self-initiated (not-instrumentally cued) behavior in freely moving animals, CNs are also transiently engaged. Our data agree with precue changes in cholinergic release that had been directly measured in the prefrontal cortex using choline-sensitive electrode at the millisecond scale, changes that had been directly correlated with sensory cue detection (Parikh et al., 2007). Hence, BF with cholinergic and non-cholinergic-projecting neurons might be an important region to participate in anticipatory behavior and improve animal performance.

### Basal Forebrain and Stimulus Discrimination

In addition to the stimulus anticipatory response, we found that BF neurons (including cholinergics) changed their activity when the CS or odor was presented. Afferents from the HDB/MCPO project to the whole olfactory system (Zaborszky, 2012) and has been proposed that GABAergic and CNs are required for proper stimulus discrimination. GABA released from BF projecting neurons into the olfactory bulb (the first brain region involved in the processing of olfactory information) is required to discriminate between similar olfactory cues, in part by inhibiting local inhibitory neurons in the bulb (Nunez-Parra et al., 2013). On the other hand it has also been proposed through *in vitro* and *in vivo* electrophysiology that ACh is required for olfactory sensory discrimination and odor memory formation (Fletcher and Chen, 2010; Chapuis and Wilson, 2013; Smith et al., 2015). At the circuitry level, it is believed that acetylcholine regulates olfactory information processing by sharpening the olfactory receptive fields of the output neuron of the olfactory bulb (Chaudhury et al., 2009; Ma and Luo, 2012) and increasing their firing frequency (Rothermel et al., 2014). At the level of the olfactory cortex, acetylcholine has been implicated in increasing pattern separation (Chapuis and Wilson, 2013) and increasing synchronization in the neuronal output of the bulb, which could lead to a more robust and stable learned olfactory representations in the olfactory cortex (de Almeida et al., 2013). Supporting this idea, we found that when animals were trained in a go/go task, it engaged significantly lower BF neurons during the start of the trial and CS presentation.

In other brain regions, cortically implanted choline-sensitive electrode recording in animals performing instrument-initiated detection of a light cue demonstrated that cholinergic neurotransmission is regulated with transient increase within seconds following cue detection superimposed over slower changes in cholinergic activity (Parikh et al., 2007). These transients are thought to be required for proper cue detection and behavioral output (Gritton et al., 2016). For instance, optogenetic regulation of BF CNs elicits fast modulation of neuronal activity in visual cortex, enhancing perception in mice responding to grating orientation (Pinto et al., 2013). Interestingly, in a cue-initiated auditory detection task, optogenetically identified CNs in the BF responded with changes in neuronal activity a few ms after receipt of reward or punishment (Hangya et al., 2015) and not to any other epoch of the behavioral trial, such as stimulus discrimination. Therefore, depending on the behavioral context, there appears to be differences in the dynamics of cholinergic release.

Finally, we found that a substantial number of non-cholinergic BF neurons, but not CNs, responded to water reinforcement. Our finding is consistent with a study that showed that primary reinforcement elicited robust bursting in non-CNs in a go/no–go task initiated by a tone where the animals were freely moving (Lin and Nicolelis, 2008).

In summary, we found that in a self-initiated task, BF cholinergic and non-CNs play a role in decision-making and stimulus discrimination. The behavioral response is in part correlated with BF anticipatory precue activity, which opens new targets and time windows to modulate attention. Finally, our data position the BF as a potential information integrator and a common neuronal pathway to elicit a context-adequate behavioral response.

### Speculation on the Role of Basal Forebrain Modulation on Selective Attention in Olfaction and Vision

What is the role of BF neuron modulation of early sensory processing in the olfactory and visual systems? In the visual system, optogenetic activation of BF CNs increases behavioral performance for mice engaged in the discrimination of vertical vs. horizontal drifting gratings (Pinto et al., 2013). Interestingly, cholinergic BF stimulation decreases neuronal synchronized of low-frequency oscillations (1–5 Hz) and increases the power of high-frequency gamma oscillations (60–100 Hz). In the olfactory system, chemogenetic inhibition of GABAergic BF modulation of granule cells in the olfactory bulb produced a reversible impairment in the discrimination of structurally similar odors (Nunez-Parra et al., 2013). Optogenetic stimulation of GABAergic BF inputs to olfactory bulb granule cells produces reliable inhibition of these interneurons (Nunez-Parra et al., 2013) that are key in generating gamma frequency oscillations generating synchronized gamma bursts that efficiently stimulate piriform cortex recurrent circuits that transmit the olfactory information in concentration-invariant odor coding (Schoppa, 2006; Poo and Isaacson, 2011; Bolding and Franks, 2018). The regulation of gamma oscillations by BF input in these sensory systems raises the question whether BF regulates transmission of information through phase amplitude coupling (PAC) mediating selective attention to specific stimuli (Nobre and van Ede, 2018).

Phase amplitude coupling is defined as gamma bursts of information firing at specific phases of low-frequency theta oscillations (4–12 Hz) (Soltesz et al., 1993; Lisman and Idiart, 1995; Chrobak and Buzsaki, 1998). Theta are the most global oscillations in the brain that act as a timekeeper (Siegle and Wilson, 2014) and are coherent across numerous cortical and subcortical structures arguing for its role in transfer of discrete chunks of information (Buzsaki, 2002; Tort et al., 2010). In the olfactory bulb, contextual odorant identity (is the odorant rewarded?) can be decoded from peak theta-phase referenced power of gamma oscillations in animals proficient in odorant discrimination in the go/no–go task but not in mice that have not learned to discriminate the odorants (Losacco et al., 2020) arguing for selective attention filtering of information on relevant stimuli through PAC. In the visual system of the macaque monkey, the strength of theta and of theta-rhythmic gamma modulation was markedly reduced by selective attention-altering information transfer through PAC (Spyropoulos et al., 2018). The engagement of changes in BF activity in different epochs of trials in associative learning tasks shown in this and other studies (Lin and Nicolelis, 2008; Pinto et al., 2013; Hangya et al., 2015; Devore et al., 2016; Gadziola and Wesson, 2016), and the fact that BF activity modulates oscillatory activity in olfactory bulb (Nunez-Parra et al., 2013) and visual cortex (Pinto et al., 2013), raises the question whether BF modulates selective attention within sensory systems or intermodally (Nobre and van Ede, 2018) through modulation of PAC. Whether this is the case requires future studies in the visual and olfactory system of BF regulation of PAC, stimulus decoding by phase-referenced power, and changes in behavioral accuracy by alteration of PAC by modulation of BF activity.

## Materials and Methods

### Animals

All procedures and experiments were approved by the Institutional Care and Use Committee at the University of Colorado Anschutz Medical Campus in accordance with NIH standards. We used 2- to 6-month-old mice from the Jackson Laboratories bred in-house. Mice were kept with water and food *ad libitum* in a reversed 12 h light cycle, except that, when they were trained for awake behaving recording, they were water restricted (below). To selectively express ChR2 in CNs, we used ChAT-EYFP-ChR2 mice generated by crossing ChAT-Cre mice [B6;129S6-*Chat*^*TM* 2(cre)Lowl^/J, RRID:IMSR_JAX:006410] with Rosa26-floxed-ChR2-EYFP animals [B6;129S-*Gt(ROSA) 26Sor*^*TM*32(CAG–COP4*H134R/EYFP)Hze^/J, RRID:IMSR_JAX:012569]. The generated mouse selectively expresses channelrhodopsin 2 (ChR2) under the control of the ChAT promoter.

### Optetrode Building

Optetrodes were built as previously shown with custom modifications described in Li et al. (2014). Briefly, four tetrodes consisting of four polymide-coated nichrome wires (diameter, 12.5 μm; Sandvik) were connected to a 16-channel interface board (EIB-16, Neuralynx) and fed through a housing glued to the board. An optic fiber (105 μm diameter, Thor Labs) was also fed through the housing, and the tetrode tips were glued maximizing the distance between them to the end of the bare fiber. Immediately before implantation, the tetrodes were gold plated to an impedance of 200–350 MΩ.

### Surgery

Adult mice were anesthetized with an intraperitoneal injection of ketamine (100 mg/kg) and xylazine (10 mg/kg). Mice were implanted in the BF at coordinates of anterior–posterior (AP) of 0.02 mm and medial–lateral (ML) of −1.625 mm, or AP of 1 mm and ML of −1.500 mm with respect to bregma. On the day of the surgery, the optetrode was implanted 200 μm above the final location, and every day, it was lowered to 50 μm until reaching a final depth of dorsal–ventral (DV) of 5 and 4.9 mm, respectively. A screw was also implanted in the skull in the opposite hemisphere (1 mm right and 2 mm posterior of bregma) to serve as ground connector. Light was delivered through the fiber, and recordings were made in order to verify neuronal light responses. The animals were allowed to recover at least 1 week before experiments were performed. Implant location was corroborated through CT scan imaging.

### Non-invasive Micro-CT Imaging

All CT imaging protocols were developed at the Animal Imaging Shared Resources (AISR) supported by the University of Colorado Cancer Center (NCI P30CA046934) and the Colorado Clinical and Translational Sciences Institute (NIH/NCATS UL1TR001082). Mice were anesthetized with 2% isoflurane, placed on a warming pad, and inserted into a Siemens Inveon micro-CT scanner (Siemens Preclinical Solutions). A single 3-dimensional (3D) micro-CT image set was acquired for each mouse using Inveon Acquisition Workstation software (IRW v1.5) with the following parameters: 270_ rotation; 240 rotation steps; charge-coupled device (CCD) readout of 2,304/2,048; 4 binnings for matrix size reduction; exposure time of 30 ms with 80 kV voltage and 450 mA current; with a field of view (FOV) of 30 mm. The 6-min acquisition with middle-to-high magnification resulted in effective isotropic resolution of 54 μm (after the Shepp–Logan reconstruction algorithm). Animals were monitored during recovery from the anesthesia and returned to their cages. The images were read with the RadiAnt DICOM Viewer 1.9.16, and measurements were made from the tip of the electrode to the dorsal, ventral, and medial aspect of the skull, taken in the coronal, sagittal, and horizontal view. With this measurements, the CT scan images were registered into an MRI atlas (AtlasView 1.0, Radiology Department Johns Hopkins University) and finally into the Paxinos Mouse Brain Atlas (George Paxinos, 2001). Eight out of 14 animals with correct implant locations were considered in the study.

### Behavior

We used instrumental conditioning in freely moving mice in the Slotnick olfactometer (Slotnick and Restrepo, 2005; Doucette et al., 2011). Animals were trained in the go/no–go and go–go behavioral task as explained in detail in Doucette et al. (2011) and Li et al. (2015). Briefly, thirsty animals were trained to discriminate between a rewarded (CS^+^) and unrewarded (CS^–^) odor. Each trial was freely initiated by the mouse entering the odor port and breaking a photodiode beam. Once the trial was started (tstart) 1–1.5 s later, the CS was presented for 2.5 s (Figure 1A). After CS delivery, the animal had to stay in the odor port for at least 500 ms for a trial to be considered completed. If not, it was considered a premature exit, and the trial had to be started again. During CS presentation, the animal learned to lick onto the water port at least once in four 0.5-s segments in response to CS^+^ for a 10-μl water reward. They quickly learned to refrain from licking in response to CS^–^ since no water was rewarded. The animal’s performance was evaluated in blocks (maximum of 10 blocks) of 20 trials (10 rewarded and 10 unrewarded, presented at random). Each block’s percent correct value represents the percent of trials in which the odors were correctly discriminated and associated with the appropriate behavioral action. Each session included 4–10 blocks of 20 trials. Electrophysiological recordings of the segments where the animal reached criteria (80% of correct responses) were considered in this study. For the go–go task, mice were rewarded at random in 70% of the trials regardless of which of the two odors was presented. The odors used were isoamylacetate, phenylacetate, 2-butatnone, ethyl propionate, ethyl butyrate, and mineral oil, all diluted at 1% in mineral oil. Experiments were performed in the afternoon (1–5 PM) under the “light on” cycle.

### Electrophysiological Recordings and Spike Clustering

The output of the tetrodes was connected to a 16-channel amplifier (A-M Systems 3500) through a 1× gain headstage (Tucker-Davis Technologies). The signal was amplified 1,000× and was recorded digitally at 24 kHz with a Data Translation DT3010 A/D card in a PC computer controlled with a custom MATLAB (Mathworks) program. Behavioral epochs or events (tstart, CS presentation, water delivery) were also recorded by the A/D board in real time.

The spike clustering method was explained in detail in Li et al. (2015) Briefly, data were filtered digitally between 300 and 3,000 Hz. With custom-written MATLAB programs, each of the 16 channels was thresholded at three times the standard deviation of the mean. Every spike with amplitude bigger than the threshold was imported into a second program (1 ms record per spike) that performed superparamagnetic clustering and wavelet decomposition of the spikes using 13 different wavelets and three principal components for the analysis and previously described (Doucette et al., 2011; Li et al., 2015). A single unit was defined as a unit with a refractory period of 1 ms (Jeanne et al., 2013; Stubblefield et al., 2015) and a violation <2% in the inter spike interval (ISI). Data for multi- and single units were used for analysis. For the go/no–go task, we found that out of 156 total units, 141 were single units. In the case of the go–go task, we registered from 1 multiunit and 43 single units. Identified CNs were all single units (Supplementary Figure S1).

### Confocal Imaging

To visualize ChR2-EYFP expression, mice were intracardially perfused with 4% paraformaldehyde and the brains postfixed overnight in the same fixative at 4°C. Thereafter, the brains were placed in a sucrose solution [30% in phosphate-buffered saline (PBS)] until they sank in the solution. Subsequently, they were frozen in dry ice and stored at −80°C. The brains were sliced at 40 μm in a cryostat, mounted on slides, and visualized with a Leica SP5 X confocal microscope.

### Delivery of Light Stimulus

A light pulse protocol was delivered to ChAT-ChR2 mice after a successful behavioral session for optogenetic tagging of CNs. A 473-nm blue laser (Shanghai Laser) was used with a maximal power of 5.3 mW (66.3 mW/mm^2^) measured at the end of the fiber under steady illumination. In the same chamber where the behavior was performed, we delivered 10 pulses of 50-ms duration at a frequency of 5 Hz. The light delivery protocol was repeated 10 times, and only the first pulse on each trial was considered for analysis.

### Slice Preparation for *in vitro* Whole Cell

Choline acetyl transferase-ChR2 mice (2–3 months old) were anesthetized by CO_2_ inhalation and decapitated. Brains were quickly removed and placed in ice-cold oxygenated sucrose slicing solution composed of (in mM): 234 sucrose, 11 glucose, 26 NaHCO_3_, 2.5 KCl, 1.25 NaH_2_PO_4_ 10, MgSO_4_, and 0.5 CaCl_2_ (equilibrated with 95% O_2_ and 5% CO_2_, pH 7.4). Coronal brain slices (300-μm thickness) were prepared using a Leica VT1200S vibratome (Leica Biosystems). Coronal slices were incubated in prewarmed (36°C), oxygenated artificial cerebrospinal fluid (ACSF; in mM): 126 NaCl, 26 NaHCO_3_, 10 glucose, 2.5 KCl, 1.25 NaH_2_PO_4_, 2 MgCl_2_, and 2 CaCl_2_ for at least 30 min before being transferred to the recording chamber, where they will be continuously perfused with ACSF (32°C).

### Whole Cell Recording

Positive and negative ChAT-ChR2 neurons in the BF (HDB/MCPO) were visually identified by EYFP expression and differential interference contrast (DIC) on a modified Olympus upright microscope (Scientifca, East Sussex, United Kingdom). Whole cell recording was performed with a Multiclamp 700B amplifier (Molecular Devices Corp.), using recording pipettes with resistance of 3–5 MΩ pulled on a PC10 vertical puller (Narishige International) and filled with intracellular solution containing the following (in mM): 135 potassium gluconate, 20 KCl, 10 HEPES, 0.1 ethylene glycol tetraacetic acid (EGTA), 2 MgATP, and 0.3 NaGTP. Recordings were low-pass filtered at 4 kHz (Bessel filter) and digitized at 10 kHz (Digidata 1440) using pClamp 10.3 software (Molecular Devices Corp.). Series resistances were monitored throughout each voltage-clamp recording with 50 ms and −10 mV steps, and if it changed by >20%, the data were discarded. Evoked synaptic responses were recorded from ChAT^+^ and ChAT^–^ neurons, and these responses were triggered by light stimulation directly onto the BF area. Light stimulation was evoked by a single mercury-free LED illumination system (CoolED pE-100 series) at 470 nm for 5 ms between 1 and 5% of the maximal intensity of the system. Latencies of evoked responses were analyzed using prewritten code routines in Axograph-X.

### Data Analysis

To determine the responsiveness of the units to the different events, we aligned all trials to the starting point of the event and calculated the average FR (in Hz). We performed a paired *t* test between the FR 1 s before and after the event and corrected the *p* value for multiple comparisons using the false discovery rate (Curran-Everett, 2000). To display the results, the FR was calculated in 0.1-s bins and normalized per unit to the mean FR, 1.2 s before the beginning of the event for 1 s. To calculate the first bin of responsiveness, we determined the first bin that exhibited a change in normalized FR (either increase or decrease) two times above or below the standard deviation of the mean with a sliding window of six bins used as a baseline.

To determine the latency of the response *in vitro* in current clamp mode, the mean latency between the beginning of the light pulse to the peak of the voltage change was measured for 15 trials. For *in vivo* recordings, the mean latency was calculated for 10 trials and defined as the time a spike was detected after the light stimulation and before 200 ms (were another light pulse was given). To identify CNs, the recordings obtained during the behavior and light delivery were processed in batch, and the same units were identified in both recordings. We calculated the latency of the first spike after the first light pulse with a custom program written in MATLAB (Mathworks), with the average of 10 trials defined as the light latency for the unit. To calculate the reliability of the response, a spike had to occur at least once 200 ms before the light was applied, and for 100% reliability, it had to spike during that period of time on each of the 10 trials. To calculate the changes in FR, we calculated the peristimulus time histogram (PSTH) of the 10 trials and performed a pairwise *t* test between baseline (500 ms–1 s) and 30 ms postlight application. The *p* value obtained for all the units was corrected for multiple comparisons (Curran-Everett, 2000). Extracellular recording from the electrodes was used to calculate the local field potential (LFP) in the frequency range from 1 to 100 Hz. Time–frequency power decomposition of the LFP was obtained by means of MATLAB’s spectrogram.m function with a 1-s window and 90% overlap. To compare LFP power between genotypes, we utilized Mann–Whitney *U* test with false discovery rate correction for multiple comparisons (Curran-Everett, 2000).

## Data Availability Statement

The datasets generated for this study are available on request to the corresponding author.

## Ethics Statement

The animal study was reviewed and approved by the Institutional Animal Care and Use Committee University of Colorado Anschutz Medical Campus.

## Author Contributions

AN-P and DR designed the *in vivo* experiments. AN-P, CC-DR, and MH designed the *in vitro* experiments. AN-P performed the *in vivo* experiments. CC-DR performed the *in vitro* experiments. AN-P and DR performed the data analysis. All authors wrote the manuscript.

## Conflict of Interest

The authors declare that the research was conducted in the absence of any commercial or financial relationships that could be construed as a potential conflict of interest.

## Supplementary Material

FIGURE S1 **(A)** Implant location was determined through CT scan imaging (Siemens Inveon animal CT scanner) and posterior electrode registration onto the Paxinos Mouse Brain Atlas **(B)**. The tetrodes can be observed in the BF in the coronal (white arrow) and sagittal CT images (bottom). The resolution of the horizontal CT allows to individually identify single tetrodes (top inset). 10 out of 16 animals were correctly implanted in the BF and included in this study. **(C)** Example of cluster analysis of one tetrode and one session. The spikes features, waveform, cluster size and inter spike interval (ISI) can be observed for a multi-unit (red) and a single unit (blue). **(D)** Bar histogram for all the units recorded in the go/no–go task *in vivo*. Top, single units, bottom, multi units.

FIGURE S2 **(A)** Heatmap of the normalized FR of all the units recorded during a Go/noGo task sorted by the delta FR between 1 s before and 1 s after trial start. During a Go/noGo task 24.2 and 25.3% of units responded to tstart during HIT and CR trials, respectively. Out of the 67 units that were recorded during FA trials, only 5,9% changed their FR in response to the tstart. **(B)** Heat map of the normalized FR of all the neuronas recorded in the Go/Go task. Units that responded to tstart = 18.2%, CS presentation = 9.1% and reward = 4.8% (Chi squared, *p* < 0.05, corrected for multiple comparisons). **(C)** Comparison of the percentage of neurons responding to tstart, CS and reward presentation between the Go/noGo and Go/Go task. Statistical significance was determined by a Chi squared corrected for multiple comparisons (*p* < pFDR = 0.0278, the correction was applied at the same time to the graph in Figure 1E). **(D)** Table depicting the responsiveness of all the neurons recorded in the Go/noGo task, sorted by the change in FR exhibited during tstart. Notice that a large percentage of these cells (44.9%) did not change their FR significantly in any of the trial epochs, while 6.1% exhibited responses in all epochs. Out of the 31 units that exhibited a statistical decrease in FR during the stimulus presentation, 14 showed a previous increase during trial initialization, suggesting that previous neuronal activity could affect changes in FR later in the trial. However, 15 additional units showing an increase in FR during the odor epoch, exhibited no change in FR during tstart and two had a decrease in FR in response to trial initialization. In the other hand, out of the 12 units that exhibited an increase in FR during the stimulus presentation, 4 also showed an increase during trial initialization, three a decrease during trial initialization, and 5 had no change during this epoch.

FIGURE S3 **(A)** Cholinergic units exhibited, in addition to a latency of the first spike after light stimulation smaller than 10 ms, a significant increase in FR after light stimulation (*t*-test, *p* < pFDR = 0.0062, corrected for multiple comparisons) and a reliability of response of 100% **(B)**. **(C)** Cholinergic neurons also exhibited low jitter (mean 4.9 ms). **(D)** Example of a cholinergic neuron responding at trial initialization (tstart). All the trials are aligned to tstart (time = 0 s, dashed black line) and sorted by odor presentation (orange line). **(E)** Top: Example of a cholinergic neuron that did not respond to reward. The PSTH was aligned to reward. Bottom: summary of the normalized FR responses to reward of all the identified cholinergic neurons (*n* = 6). Even though there appears to be a disturbance in the FR a few ms after time = 0, the changes are not statistically significant.
