# Supplementary figures and images for: The Basal Forebrain Modulates Neuronal Response in an Active Olfactory Discrimination Task
Source: Front Cell Neurosci. 2020 Jun 5;14:141. doi: 10.3389/fncel.2020.00141 (PMC7289987; doi:10.3389/fncel.2020.00141)

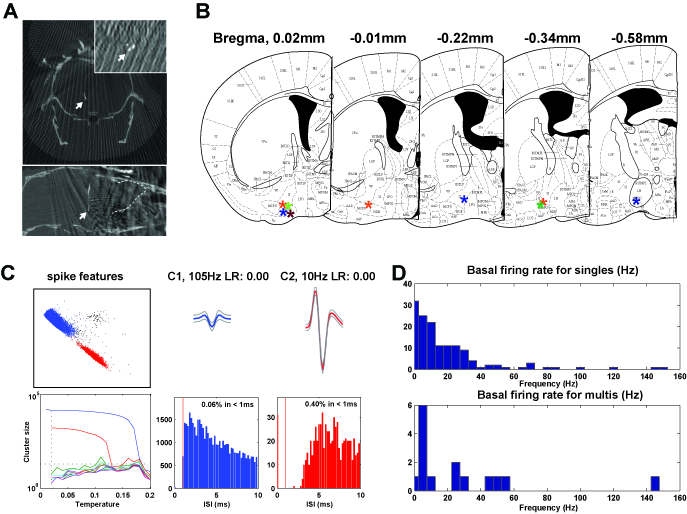

Supplement: FIGURE S1 — (A) Implant location was determined through CT scan imaging (Siemens Inveon animal CT scanner) and posterior electrode registration onto the Paxinos Mouse Brain Atlas (B). The tetrodes can be observed in the BF in the coronal (white arrow) and sagittal CT images (bottom). The resolution of the horizontal CT allows to individually identify single tetrodes (top inset). 10 out of 16 animals were correctly implanted in the BF and included in this study. (C) Example of cluster analysis of one tetrode and one session. The spikes features, waveform, cluster size and inter spike interval (ISI) can be observed for a multi-unit (red) and a single unit (blue). (D) Bar histogram for all the units recorded in the go/no–go task in vivo. Top, single units, bottom, multi units. [file Image_1.tif]

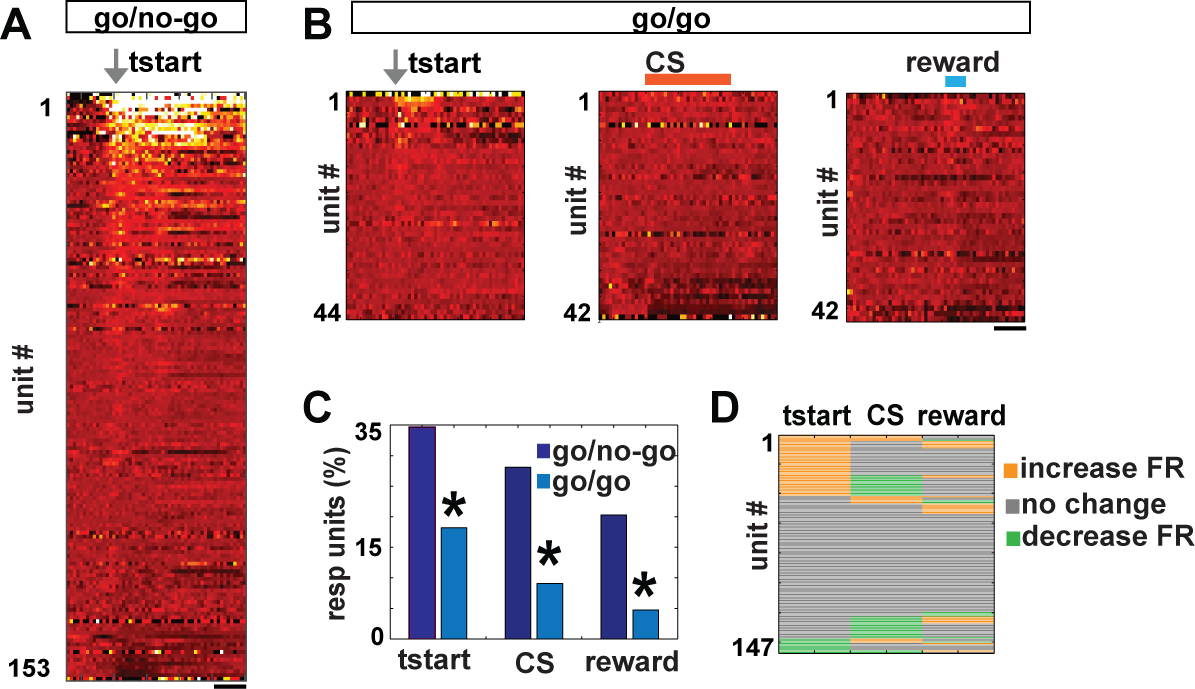

Supplement: FIGURE S2 — (A) Heatmap of the normalized FR of all the units recorded during a Go/noGo task sorted by the delta FR between 1 s before and 1 s after trial start. During a Go/noGo task 24.2 and 25.3% of units responded to tstart during HIT and CR trials, respectively. Out of the 67 units that were recorded during FA trials, only 5,9% changed their FR in response to the tstart. (B) Heat map of the normalized FR of all the neuronas recorded in the Go/Go task. Units that responded to tstart = 18.2%, CS presentation = 9.1% and reward = 4.8% (Chi squared, p < 0.05, corrected for multiple comparisons). (C) Comparison of the percentage of neurons responding to tstart, CS and reward presentation between the Go/noGo and Go/Go task. Statistical significance was determined by a Chi squared corrected for multiple comparisons (p < pFDR = 0.0278, the correction was applied at the same time to the graph in Figure 1E). (D) Table depicting the responsiveness of all the neurons recorded in the Go/noGo task, sorted by the change in FR exhibited during tstart. Notice that a large percentage of these cells (44.9%) did not change their FR significantly in any of the trial epochs, while 6.1% exhibited responses in all epochs. Out of the 31 units that exhibited a statistical decrease in FR during the stimulus presentation, 14 showed a previous increase during trial initialization, suggesting that previous neuronal activity could affect changes in FR later in the trial. However, 15 additional units showing an increase in FR during the odor epoch, exhibited no change in FR during tstart and two had a decrease in FR in response to trial initialization. In the other hand, out of the 12 units that exhibited an increase in FR during the stimulus presentation, 4 also showed an increase during trial initialization, three a decrease during trial initialization, and 5 had no change during this epoch. [file Image_2.tif]

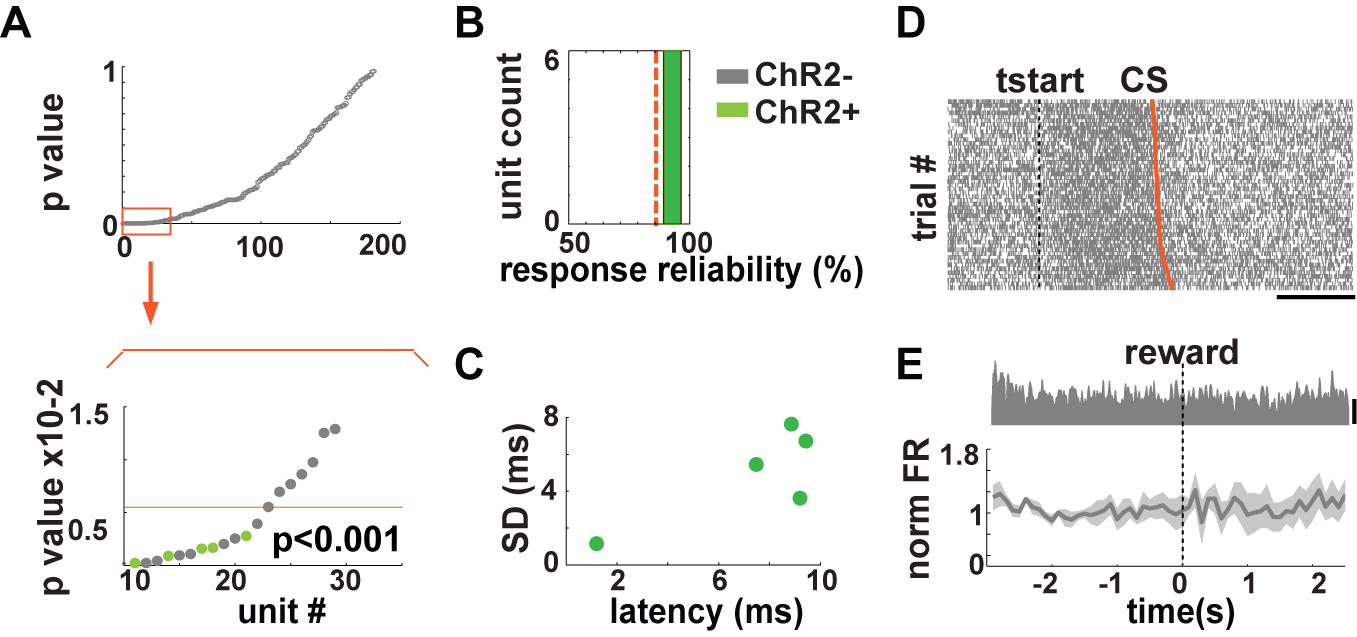

Supplement: FIGURE S3 — (A) Cholinergic units exhibited, in addition to a latency of the first spike after light stimulation smaller than 10 ms, a significant increase in FR after light stimulation (t-test, p < pFDR = 0.0062, corrected for multiple comparisons) and a reliability of response of 100% (B). (C) Cholinergic neurons also exhibited low jitter (mean 4.9 ms). (D) Example of a cholinergic neuron responding at trial initialization (tstart). All the trials are aligned to tstart (time = 0 s, dashed black line) and sorted by odor presentation (orange line). (E) Top: Example of a cholinergic neuron that did not respond to reward. The PSTH was aligned to reward. Bottom: summary of the normalized FR responses to reward of all the identified cholinergic neurons (n = 6). Even though there appears to be a disturbance in the FR a few ms after time = 0, the changes are not statistically significant. [file Image_3.tif]
